# In Silico Pharmacology for Evidence-Based and Precision Medicine

**DOI:** 10.3390/pharmaceutics15031014

**Published:** 2023-03-22

**Authors:** Marios Spanakis

**Affiliations:** 1 Department of Forensic Sciences and Toxicology, Faculty of Medicine, University of Crete, GR-71003 Heraklion, Greece; marspan@ics.forth.gr; 2 Computational BioMedicine Laboratory, Institute of Computer Science, Foundation for Research & Technology—Hellas, GR-71110 Heraklion, Greece

Personalized/precision medicine (PM) originates from the application of molecular pharmacology in clinical practice, representing a new era in healthcare that aims to identify and predict optimum treatment outcomes for a patient or a cohort with similar genotype/phenotype characteristics. Evidence-based medicine (EBM) integrates accumulated information from basic in vitro, in vivo, and observational studies as well as clinical trials and systematic review meta-analysis data for clinical consideration for a population. Hence, it can be stated that EBM often sees the forest (population averages) for the trees (individual patients), whereas the utilization of PM may not see the forest for the trees [1]. In either case, in recent years, the utilization of the digital revolution in pharmaceutical research has led to the emergence of innovative approaches and disciplines such as pharmacometrics and quantitative systems pharmacology (QSP) that combine experimental and/or clinical data to predict and/or interpret the pharmacological profiles of molecules of interest considering complex associations of biological pathways, disease, and physiological characteristics and their variabilities within patient cohorts [2,3]. These state-of-the-art tools of modelling and simulation (M&S) in pharmacology try to extrapolate knowledge gained through experimental and clinical procedures through either top-down or bottom-up approaches of potential new drug targets or the role of specific biological molecules in disease initiation and progression. In addition, they contribute to the R&D of novel drug-delivery systems and greatly assist in drug repurposing. Hence, they provide sophisticated biomedical tools covering a broad range of studies on the research and development (R&D) of novel, more efficient, and more effective molecules, with improved safety profiles for a patient concerning PM principles and greater chances to proceed in clinical trials, enhancing the knowledge within the EBM hierarchy.

The application of advanced quantitative structure–activity relationship-based (QSAR) studies can provide insights into potential mechanisms or binding positions in targeted proteins along with physicochemical requirements for candidate drugs. This can assist in the discovery of molecules with desired or enhanced modulatory activity against targeted proteins by a well-organized screening of compound databases such as plants’ secondary metabolites for pharmacologically active compounds and potential new drug candidates [4]. In addition, through ligand-based methods, classification models and molecular docking simulations for possible pharmacological properties, sophisticated virtual screening frameworks assist in drug repurposing studies of already-marketed drugs such as the example of modulators of transient receptor potential vanilloid-1 (TRPV1) in analgesia, or for possible effective therapies in diseases with an apparent need for treatment options (i.e., COVID-19) [5,6]. Expanding the QSAR approaches, the combination of in silico pharmacology tools with experimental in vitro protocols can provide new insights regarding drugs actions and potential secondary mechanisms that could be related to adverse drug reactions (ADRs). This is important for drugs that are considered essential in medicine where the associated ADR mechanisms are partially explained at a molecular level. For example, the combination of in silico tools with in vitro systems allowed the generation of a potential molecular explanation of how zoledronic acid binds to the inward rectifying potassium (Kir) channel subunit KIR6.1/6.2 and sulfonylurea receptor subunits (SUR2A/B) and blocks the ATP-sensitive potassium (KATP) channels reducing native currents in the fibres and bone cells [7]. These findings can be further exploited to interpret molecular mechanisms in frequently reported ADRs regarding the occurring arrhythmias or other effects related to zoledronic acid administration.

Nevertheless, on top of all in silico approaches, one area that in previous years represented a sea change in pharmacology, transformed R&D forever, and brought in silico pharmacology into the limelight of modern biomedical research is the pharmacokinetic (PK) and pharmacodynamic (PD) M&S, such as the physiologically based pharmacokinetic (PBPK) models and pharmacometrics [8]. These approaches are capable of integrating drug, disease, and experimental data along with clinical information towards optimum efficacy and safety profiles of drugs in clinical settings [9]. For instance, the utilization of PK models can sufficiently predict PK profiles of drugs with narrow therapeutic indexes and/or of high intra- or inter- subject variability in general or in special population groups. For example, through pharmacometrics approaches, the PK profile of busulfan administration in paediatric patients undergoing hematopoietic stem cell transplantation (HSCT) can be utilized for an optimum dosing regimen [10]. In addition, even for drugs marketed for a long time (i.e., nebivolol), population–PK model approaches can provide important information through the M&S of different scenarios such as the impact of age or genotype characteristics on optimum dose selection regarding drugs’ efficacy and safety [11]. Regarding PBPK models and population PK simulations, they can deliver essential information with the generation of in silico clinical trials and provide mechanistic insights, the characterization of drugs’ variability for different clinical scenarios, or different drug formulations for clinical applications [12,13]. For example, through PBPK M&S, possible therapeutic peculiarities related to doxorubicin administration and its metabolites in the body can be simulated and studied under different scenarios [14]. Moreover, developed PBPK models for drugs such as ropinirole can be further utilized to predict the exposure of new prolonged-release formulations for oral administration in various doses considering in vitro/in vivo extrapolated dissolution data [15]. It is important to point out that in order to achieve successful application of PBPK M&S, the detailed depiction of the three major components that contribute to these approaches are necessary: (i) system-specific properties (organ composition, variability among individuals, body fluid characteristics, etc.), (ii) drug properties (physicochemical or biochemical properties, etc.), and (iii) trial design (target population, route of administration, formulation, etc.). Thus, studies that describe essential parameters such as organ properties are always needed. For example, PBPK models that consider the bidirectional and site-dependent cerebrospinal fluid (CSF) movement can be further utilized in the accurate prediction of PK profiles of small molecules after intra-CSF administration [16].

An additional advantage of the continuously evolving in silico pharmacology frameworks is that they offer a low-cost, quick, and systematic high-throughput approach to guide the prioritization of targets and identify critical molecular markers for various diseases, especially those with complex molecular mechanisms, e.g., schizophrenia/bipolar disorder, rheumatoid arthritis, diabetes mellitus, hypertension, Alzheimer’s disease, cancer, etc. This fact, combined with the exploitation of algorithms that can “learn” patterns within a set of classified data and their interconnections to make predictions, suggestions, or decisions, creates a sea of possibilities for the next era in drug research and modern pharmacology [17]. For example, bioengineering approaches that utilize learning algorithms such as machine learning for transcriptomic data can analyse gene–disease associations to identify potential drug targets, whereas coupling these results with QSAR models can create a research field for identifying molecules with potential pharmacological activity for future drug development [18,19].

Overall, computational pharmacology M&S approaches have advanced well beyond the state-of-the-art of being simple research tools and not only achieved an active role in the R&D of novel medicinal products but also received regulatory acceptance [8,20]. It is expected that the further utilization of in silico pharmacology tools integrated with artificial intelligence algorithms will further remove barriers and obstacles regarding our understanding of the complex interplay between drugs, targets, and diseases. Therefore, it is expected that computational or in silico pharmacology within the context of quantitative systems pharmacology will allow us to further “connect the dots” and reveal the bigger picture, the “population/forest” that will assist EBM decisions, considering what kind of “individuals/trees” exist within a patient cohort according to PM principles.

## References

[1] Spanakis M., Patelarou A.E., Patelarou E. (2020). Nursing Personnel in the Era of Personalized Healthcare in Clinical Practice. J. Pers. Med..

[2] Standing J.F. (2017). Understanding and applying pharmacometric modelling and simulation in clinical practice and research. Br. J. Clin. Pharmacol..

[3] Knight-Schrijver V.R., Chelliah V., Cucurull-Sanchez L., Le Novère N. (2016). The promises of quantitative systems pharmacology modelling for drug development. Comput. Struct. Biotechnol. J..

[4] Aribisala J.O., Sabiu S. (2022). Cheminformatics Identification of Phenolics as Modulators of Penicillin-Binding Protein 2a of Staphylococcus aureus: A Structure–Activity-Relationship-Based Study. Pharmaceutics.

[5] Andrei C., Mihai D.P., Zanfirescu A., Nitulescu G.M., Negres S. (2022). In Silico Drug Repurposing Framework Predicts Repaglinide, Agomelatine and Protokylol as TRPV1 Modulators with Analgesic Activity. Pharmaceutics.

[6] Raman A.P.S., Kumari K., Jain P., Vishvakarma V.K., Kumar A., Kaushik N., Choi E.H., Kaushik N.K., Singh P. (2022). In Silico Evaluation of Binding of 2-Deoxy-D-Glucose with Mpro of nCoV to Combat COVID-19. Pharmaceutics.

[7] Maqoud F., Scala R., Tragni V., Pierri C.L., Perrone M.G., Scilimati A., Tricarico D. (2021). Zoledronic Acid as a Novel Dual Blocker of KIR6.1/2-SUR2 Subunits of ATP-Sensitive K+ Channels: Role in the Adverse Drug Reactions. Pharmaceutics.

[8] Jamei M. (2016). Recent Advances in Development and Application of Physiologically-Based Pharmacokinetic (PBPK) Models: A Transition from Academic Curiosity to Regulatory Acceptance. Curr. Pharmacol. Rep..

[9] Marshall S.F., Burghaus R., Cosson V., Cheung S., Chenel M., DellaPasqua O., Frey N., Hamrén B., Harnisch L., Ivanow F. (2016). Good Practices in Model-Informed Drug Discovery and Development: Practice, Application, and Documentation. CPT Pharmacomet. Syst. Pharmacol..

[10] Neroutsos E., Nalda-Molina R., Paisiou A., Zisaki K., Goussetis E., Spyridonidis A., Kitra V., Grafakos S., Valsami G., Dokoumetzidis A. (2022). Development of a Population Pharmacokinetic Model of Busulfan in Children and Evaluation of Different Sampling Schedules for Precision Dosing. Pharmaceutics.

[11] Marques L., Costa B., Vale N. (2022). New Data for Nebivolol after In Silico PK Study: Focus on Young Patients and Dosage Regimen. Pharmaceutics.

[12] Rostami-Hodjegan A. (2012). Physiologically Based Pharmacokinetics Joined With In Vitro–In Vivo Extrapolation of ADME: A Marriage Under the Arch of Systems Pharmacology. Clin. Pharmacol. Ther..

[13] Vizirianakis I.S., Mystridis G.A., Avgoustakis K., Fatouros D.G., Spanakis M. (2016). Enabling personalized cancer medicine decisions: The challenging pharmacological approach of PBPK models for nanomedicine and pharmacogenomics (Review). Oncol. Rep..

[14] Mystridis G.A., Batzias G.C., Vizirianakis I.S. (2022). Physiologically Based Pharmacokinetic Modelling and Simulation to Predict the Plasma Concentration Profile of Doxorubicin. Pharmaceutics.

[15] Shuklinova O., Dorożyński P., Kulinowski P., Polak S. (2022). Quality Control Dissolution Data Is Biopredictive for a Modified Release Ropinirole Formulation: Virtual Experiment with the Use of Re-Developed and Verified PBPK Model. Pharmaceutics.

[16] Hirasawa M., de Lange E.C.M. (2022). Revisiting Cerebrospinal Fluid Flow Direction and Rate in Physiologically Based Pharmacokinetic Model. Pharmaceutics.

[17] Kumar M., Nguyen T.P.N., Kaur J., Singh T.G., Soni D., Singh R., Kumar P. (2023). Opportunities and challenges in application of artificial intelligence in pharmacology. Pharmacol. Rep..

[18] Wu A.T.H., Lawal B., Wei L., Wen Y.T., Tzeng D.T.W., Lo W.C. (2021). Multiomics Identification of Potential Targets for Alzheimer Disease and Antrocin as a Therapeutic Candidate. Pharmaceutics.

[19] Zhao K., Shi Y., So H.-C., Zhao K., Shi Y., So H.-C. (2022). Prediction of Drug Targets for Specific Diseases Leveraging Gene Perturbation Data: A Machine Learning Approach. Pharmaceutics.

[20] Bai J.P.F., Schmidt B.J., Gadkar K.G., Damian V., Earp J.C., Friedrich C., van der Graaf P.H., Madabushi R., Musante C.J., Naik K. (2021). FDA-Industry Scientific Exchange on assessing quantitative systems pharmacology models in clinical drug development: A meeting report, summary of challenges/gaps, and future perspective. AAPS J..

